# Recent Developments in Osteoarthritis Research: Innovative Therapeutic Approaches and the Role of Polyphenols and Nanotechnology

**DOI:** 10.3390/ijms26188925

**Published:** 2025-09-13

**Authors:** Humberto Vélez-Slimani, Jacobo Hernández-Montelongo, Luis A. Salazar

**Affiliations:** 1 Doctoral Program in Sciences Major in Applied Cellular and Molecular Biology, Universidad de La Frontera, Temuco 4811230, Chile; h.velez01@ufromail.cl; 2 Center for Molecular Biology and Pharmacogenetics, Department of Basic Sciences, Universidad de La Frontera, Temuco 4811230, Chile; 3 Department of Mathematical and Physical Sciences, Universidad Católica de Temuco, Temuco 4780000, Chile; jacobo.hernandez@uct.cl

**Keywords:** osteoarthritis, polyphenols, porous silicon, β-cyclodextrin, nanoparticles, oxidative stress, inflammation

## Abstract

Osteoarthritis (OA) is a chronic degenerative joint disease with a significant impact on quality of life. This review summarizes recent advances in the understanding of OA pathophysiology, emphasizing mechanical factors, angiogenesis, aging, and the role of interleukins in disease progression. It also explores key molecular pathways, particularly the NF-κB signaling cascade, and crucial catabolic mediators such as MMPs and ADAMTS, which are involved in cartilage degradation. Emerging therapeutic strategies are discussed, with a focus on the potential of polyphenols, such as pinocembrin and caffeic acid phenethyl ester, in cartilage protection and regeneration. In addition, nanotechnology is examined as a promising tool to enhance the bioavailability of these bioactive compounds and support the development of innovative OA treatments. Drug delivery systems based on nanotechnology, including porous silicon nanoparticles functionalized with β-cyclodextrin, have shown promise in improving therapeutic efficacy. This review highlights the importance of multidisciplinary approaches integrating molecular biology, natural compounds, and advanced technologies in the treatment of OA.

## 1. Introduction

Osteoarthritis (OA) is a degenerative joint disease characterized by the progressive deterioration of cartilage, alterations in subchondral bone, and synovial inflammation [1]. According to the 2019 Evidence-Based Guidelines for OA developed by the American College of Rheumatology and the Arthritis Foundation, approximately 302 million people worldwide are affected by this condition [2]. The pain and stiffness associated with OA significantly impair patients’ quality of life and pose a major public health challenge, particularly in developed countries [3]. OA represents a considerable burden on individuals, healthcare systems, and society due to medical costs and loss of income from work disability [4,5]. Despite its prevalence and socioeconomic impact, many patients do not receive adequate care, highlighting the need for an integrated and personalized medical approach [6]. Key contributors include the absence of disease-modifying osteoarthritis drugs, the predominantly symptomatic and short-lived benefit of current therapies, limited access to multidisciplinary care and affordability constraints, and substantial clinical heterogeneity that complicates individualized treatment [7,8,9,10,11]. Beyond mechanical wear, persistent inflammatory signals are now recognized as key drivers that disrupt the synthesis of essential extracellular matrix (ECM) components, such as type II collagen, while simultaneously promoting the expression of degradative enzymes. This adaptive cellular response can lead to redox imbalance and oxidative stress, both of which play a central role in OA progression [12].

**Figure 1:**
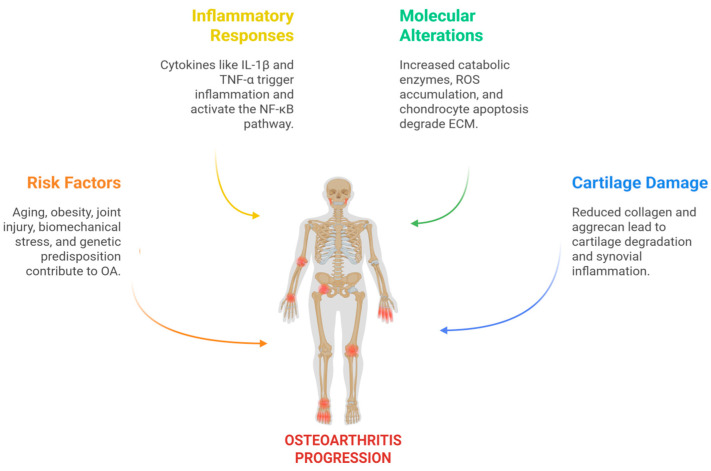
Mechanisms Involved in the Progression of OA: This diagram illustrates the progression of OA driven by multiple risk factors such as aging, obesity, joint injury, biomechanical stress, and genetic predisposition. These stimuli trigger inflammatory responses mediated by cytokines like IL-1β and TNF-α, leading to activation of the NF-κB signaling pathway. As a result, there is increased expression of catabolic enzymes such as MMP-13 and ADAMTS-5, along with the accumulation of reactive oxygen species (ROS), mitochondrial dysfunction, and chondrocyte apoptosis. These molecular alterations contribute to ECM degradation, characterized by reduced collagen type II and aggrecan, which ultimately results in cartilage damage, synovial inflammation, and subchondral bone remodeling Created in BioRender. Vélez-Slimani, H. (2025). https://BioRender.com/qmqzp3l (accessed on 9 September 2025).

OA can be classified into two main forms: primary (idiopathic), with no known cause, and secondary, which is typically associated with trauma, metabolic diseases, or other joint disorders. Moreover, different clinical and molecular phenotypes have been proposed, including inflammatory, senescence-associated, pain-persistent, and metabolic syndrome-related subtypes, reflecting the disease’s heterogeneity and suggesting that personalized therapies could improve clinical outcomes [13,14]. Risk factors such as advanced age, obesity, joint injuries, biomechanical alterations, and genetic predisposition can trigger and accelerate the degeneration of articular cartilage [15]. This process involves the activation of inflammatory pathways such as NF-κB, a versatile and multifunctional transcription factor that acts as a central regulator of the inflammatory response in OA [16]. It is also associated with increased production of catabolic mediators, particularly matrix metalloproteinases (MMPs), such as MMP-13, which lead to redox imbalance, increased oxidative stress, and chondrocyte apoptosis, thereby accelerating tissue degeneration [17,18]. Under normal conditions, the ECM of cartilage, accounting for about 90% of its dry weight, maintains a balance between synthesis and degradation, which is essential for its mechanical properties. In OA, this balance shifts toward catabolism, resulting in progressive ECM degradation and damage to the articular tissue. Therefore, restoring this homeostasis by targeting inflammatory pathways and oxidative stress is key to effective OA treatment [19] (Figure 1).

**Figure 2:**
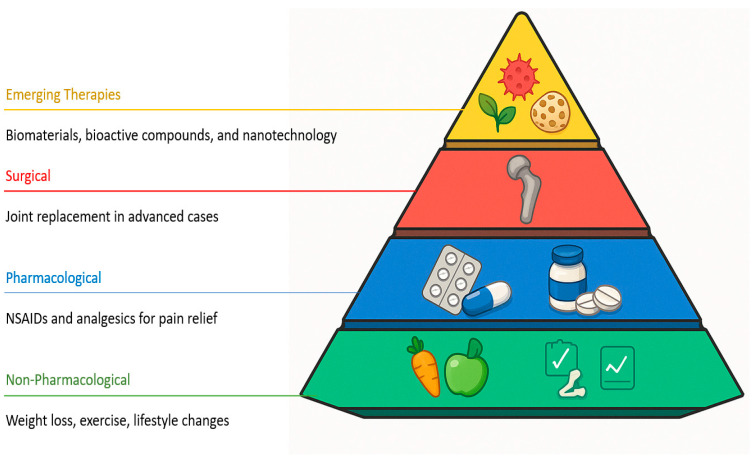
Current therapeutic strategies for OA. OA management involves pharmacological treatments such as nonsteroidal anti-inflammatory drugs (NSAIDs) and analgesics, surgical interventions including joint replacement in advanced cases, and non-pharmacological measures such as weight loss, physical exercise, and lifestyle modifications. While these approaches alleviate symptoms and improve joint function, they do not halt the structural progression of cartilage degeneration. Consequently, emerging therapies based on biomaterials, bioactive compounds, and nanotechnology aim to modulate the joint microenvironment and promote tissue repair (Created in Microsoft PowerPoint).

The treatment of OA includes a range of strategies, from pharmacological interventions to surgical options, all aimed at relieving pain and improving joint function [4]. It also includes non-pharmacological measures such as weight loss, exercise, and lifestyle modifications [20]. To date, no approved therapies have demonstrated disease-modifying effects in OA. Non-surgical approaches, including lifestyle interventions and analgesics, may provide partial symptom relief but generally fail to halt or reverse the structural progression of the disease [21]. For this reason, there is growing interest in the development of regenerative therapies based on biomaterials, bioactive compounds, and emerging technologies aimed at modulating the joint microenvironment and promoting tissue repair (Figure 2).

Polyphenols, such as curcumin, resveratrol, epigallocatechin gallate, quercetin, and propolis-derived molecules including pinocembrin and caffeic acid phenethyl ester, have attracted interest in osteoarthritis because they mitigate pro-inflammatory signaling (e.g., NF-κB), activate cytoprotective pathways (Nrf2/HO-1), and reduce catabolic mediators implicated in cartilage breakdown (e.g., MMP-13 and ADAMTS-5) [22,23,24]. However, limited aqueous solubility, instability, and rapid intra-articular clearance restrict clinical translation of many polyphenols; formulation strategies that improve bioavailability are, therefore, critical [25,26]. Nanotechnology-enabled delivery can address these barriers by enhancing solubility and retention in the joint and by enabling chondrocyte-targeted, sustained release after intra-articular administration [27,28]. Representative platforms include cyclodextrin inclusion complexes and silica–cyclodextrin hybrids, as well as porous (silica/silicon) nanoparticles, liposomes, and other polymeric/inorganic systems that support controlled release of polyphenols in the joint [29,30].

This review synthesizes advances in OA pathophysiology and molecular mechanisms, and evaluates emerging therapeutic strategies, with a special focus on polyphenols and nanotechnology. We integrate mechanistic data with nanocarrier-mediated exposure and retention, align mechanisms with clinically meaningful endpoints (pain, function, imaging), classify evidence maturity using tiered labels, and outline practical priorities to facilitate clinical translation.

## 2. Structural Homeostasis of Cartilage and Chondrocyte Activation in OA

This section provides a concise mechanistic background to frame the translational analysis that follows. We highlight the organization of the osteochondral unit and the pro-inflammatory and oxidative cascades most relevant to osteoarthritis (NF-κB activation, ROS and mitochondrial dysfunction, and MMP/ADAMTS-driven matrix loss). The osteochondral tissue exhibits a hierarchical organization composed of three interconnected layers: the articular cartilage on the surface, a transitional zone formed by calcified cartilage (CCL), and the underlying subchondral bone, which mainly consists of trabecular tissue. Within the cartilage, the structure is zonally divided into superficial, middle, and deep regions, each with a distinct cellular and ECM composition, providing specific structural and mechanical properties at each level [31]. Articular cartilage is composed of a dense ECM rich in collagen and proteoglycans, which confer its biomechanical properties [32]. Chondrocytes, the only resident cell population, exist without a blood supply or innervation and are responsible for maintaining tissue homeostasis through synthesis and turnover of ECM components [33]. Consequently, adult articular cartilage has limited intrinsic regenerative capacity and relies primarily on diffusion from synovial fluid through the extracellular matrix for nutrient delivery and waste removal [34,35,36].

The superficial zone accounts for 10–20% of the total cartilage thickness and contains thin, densely packed collagen fibers aligned parallel to the articular surface, with flattened chondrocytes tightly arranged [37]. The middle zone, comprising 40–60% of the thickness, contains thicker collagen fibers arranged in arcs, a higher concentration of glycosaminoglycans (GAGs), and rounded chondrocytes distributed randomly. Lastly, the deep zone represents 30–40% of the total thickness, with collagen fibers oriented perpendicularly to the tidemark, elongated chondrocytes, high GAG content, and low cell density. These zonal differences are essential for biomechanical function and the tissue’s response to mechanical load [38].

The calcified cartilage layer (CCL), located between deep hyaline cartilage and subchondral bone, transmits mechanical load and mediates cartilage–bone crosstalk. In OA, structural changes (tidemark duplication, thickening, microcracks) reflect failed repair, permit vascular invasion and pathological mineralization, and impair its barrier function, making the CCL a promising target for tissue-engineering approaches [39]. During OA, chondrocytes adopt a catabolic phenotype driven by altered mechanical and biochemical cues, with increased MMPs and pro-inflammatory cytokines; IL-1β is central, inducing degradative enzymes and suppressing matrix synthesis [40,41,42]. Activated chondrocytes integrate inflammatory and oxidative cues, tipping ECM turnover toward catabolism (MMP-13, ADAMTS-5) and setting up the therapeutic targets reviewed next.

## 3. Factors Contributing to the Pathophysiology of OA: Aging, Mechanics, Angiogenesis, and Genetic and Epigenetic Aspects

The prevalence of OA increases with age and is one of the leading causes of disability among older adults, driven by mechanisms such as oxidative stress and the weakening of the musculoskeletal system [43]. Aging profoundly impacts chondrocyte metabolism and homeostasis by promoting cellular senescence, mitochondrial dysfunction, chronic inflammation, and disturbances in energy metabolism [44]. In a spontaneous knee OA model, a significant reduction in ATP and mitochondrial biomass in chondrocytes has been observed, contributing to widespread metabolic dysfunction in cartilage, subchondral bone, and synovial membrane. This mitochondrial dysfunction, exacerbated by age, mechanical stress, and inflammation, increases ROS production and accelerates joint degradation [45]. Furthermore, aging reduces the ability of chondrocytes to synthesize ECM and enhances their catabolic response to inflammatory stimuli, partially mediated by the activation of the senescence-associated secretory phenotype (SASP), expression of p16^NK4a^, and dysfunction in autophagic and lysosomal processes [46].

Articular cartilage is constantly subjected to mechanical forces such as stress, strain, and pressure, which, when excessive, can cause cellular apoptosis and accelerate tissue degeneration [47]. Due to its limited self-repair capacity, cartilage defects can evolve into post-traumatic OA, where joint replacement becomes the final option at advanced stages. Although surgical interventions like microfracture or osteochondral grafts offer temporary improvements, integration with host tissue remains a major challenge. Innovative strategies, such as biological adhesives (e.g., microbial transglutaminase) and dynamic ex vivo models using human cartilage, represent promising approaches to optimize tissue repair [48]. Angiogenesis in the subchondral bone plays a critical role in OA pathogenesis. Aberrant formation of new blood vessels and nerve fibers is regulated by perivascular cells and osteoclasts, which promote bone resorption and facilitate nerve invasion, thereby accelerating cartilage degeneration and chondrocyte apoptosis [49].

Finally, genetic and epigenetic factors significantly contribute to OA susceptibility and progression. Genetic studies have identified multiple risk loci, with an estimated heritability of 60% in the hip, 40% in the knee, and up to 65% in the hands [50]. Recent investigations have characterized subchondral bone marrow lesions (BMLs) as regions with specific genetic signatures associated with nerve formation, angiogenesis, and inflammation, thereby exacerbating pain and joint damage progression [51]. In the epigenetic landscape, demethylase enzymes such as UTX, KDM7A, KDM5C, and PHF8 modulate gene expression and bone metabolism, affecting chondrocyte and osteoblast homeostasis via pathways like Wnt/β-catenin [47]. Additionally, age-related CpG site DNA methylation can induce gene silencing and promote OA. Epigenetic biomarkers such as PAI-1 and leptin have been linked to increased OA risk, particularly in the knee joint [52]. Despite their different origins, these factors converge in common mechanisms that promote ECM degradation, disrupt cartilage architecture, and drive disease progression.

Collectively, these processes are interdependent. Aging promotes chondrocyte senescence and a SASP milieu that heightens sensitivity to inflammatory cues and oxidative stress [53,54]. Abnormal mechanical loading triggers mechanotransduction pathways (integrins/FAK, PIEZO1/2, YAP/TAZ) that synergize with inflammatory signals to amplify catabolism [55,56]. Subchondral angiogenesis and sensory nerve ingrowth at the tidemark couple to osteoclast activity, facilitating structural change and pain [57]. Genetic susceptibility tunes baseline pathway activity, while epigenetic modifications stabilize pro-degenerative programs [58,59]. These interactions help explain convergence on catabolic enzymes (MMPs, ADAMTS-5) and inflammatory mediators (IL-1β, IL-6, PGE_2_, NO) [60].

## 4. Importance of Interleukin-1β in OA: Therapeutic Implications

IL-1β has been reported to destroy joint cartilages by restricting the synthesis of anabolic substances such as collagen and proteoglycans, while increasing the production of catabolic substances such as MMPs and ADAMTS, as well as promoting chondrocyte apoptosis [42]. This pro-inflammatory factor binds to specific receptors on the chondrocyte membrane, activating an intracellular cascade that includes phosphorylation and subsequent degradation of IκBα, the main inhibitor of NF-κB. This degradation, mediated by the proteasome, allows the release of the NF-κB complex, which translocates to the nucleus and promotes the transcription of pro-inflammatory and catabolic genes [61]. Recent studies have shown that chondrocytes and synovial membrane cells (synoviocytes) release the pro-inflammatory cytokine interleukin-1 (IL-1) as part of the local inflammatory response in OA. An excess of IL-1β induces chondrocytes to trigger an inflammatory reaction and matrix catabolism, accelerating ECM degradation and promoting excessive production of pro-inflammatory factors and catabolic chemicals such as PGE2, NO, COX-2 (Cyclooxygenase-2), iNOS, ADAMTS, and MMPs.

Beyond its canonical pro-inflammatory role, IL-1β signaling in OA is amplified by mechanotransduction: inflammatory cues sensitize PIEZO1-mediated Ca^2+^ influx in chondrocytes, heightening responsiveness [62]. Upstream processing via the NLRP3 inflammasome promotes caspase-1 maturation of IL-1β and can trigger pyroptosis in joint tissues [63]. Downstream, IL-1β signaling induces HIF-2α *(EPAS1)*, which drives a catabolic program including MMP-13/ADAMTS-5 and related mediators [64]. Clinically, direct IL-1 blockade has shown limited and inconsistent benefit in knee OA (e.g., lutikizumab), motivating proximal-pathway and mechano-immune strategies [65].

Several IL-1β antagonists, such as lutikizumab, show unsatisfactory results in clinical trials, highlighting the need to explore new active molecules that target IL-1β inhibition as a strategy to combat OA [65,66]. In the study developed by Lian et al. (2020), the IL-1β inhibitor showed limited results in the treatment of OA in the clinic; however, when combined with a miR-18 antagomir, better effects were obtained due to activation in TGF-β signaling and suppression of chondrocyte hypertrophy. This showed that anti-miR-18a sensitized chondrocytes with OA to the IL-1β inhibitor [67]. Therefore, the search for effective IL-1β inhibitors has become essential in the treatment of OA, offering promising therapeutic prospects [68]. Together, the pathophysiology of OA involves multiple interrelated factors from aging and mechanical loading to immune dysfunction and epigenetic reprogramming that alter the anabolic-catabolic balance of cartilage. Understanding these mechanisms is key to the development of more effective and personalized targeted therapies.

## 5. Molecular Pathways and Molecules Involved in OA

Understanding of the mechanisms underlying OA has focused on the structural and functional changes in joint tissues, as well as a variety of molecular biological processes involving various cells and cytokines. In addition, cell signaling pathways have been observed to influence their progression [69,70].

### 5.1. NF-κB Pathway in OA

The NF-κB pathway plays a key role in the pathology of OA, as it is linked to cartilage degradation, synovial inflammation, and subchondral sclerosis. It regulates the expression of genes related to degradative metabolism, such as MMPs and *ADAMTS5*, through specific response elements in their promoters, which stimulates the production of inflammatory mediators such as COX-2, PGE2, and iNOS. Its activation also induces transcription of target genes, promoting chondrocyte inflammation and apoptosis, which aggravates joint damage [71]. In OA, NF-κB is activated in response to pro-inflammatory cytokines, such as IL-1β and tumor necrosis factor (TNF)-α, as well as MMPs and prostaglandins. NF-κB has been shown to regulate these inflammatory activities and is involved in both the differentiation and destruction of cartilage, promoting the secretion of degrading enzymes and inflammatory cytokines, which contribute to joint damage [72].

NF-κB is the central pro-inflammatory hub in OA chondrocytes, driving COX-2, iNOS, MMP-13, and ADAMTS-5; yet, despite robust preclinical inhibition, randomized trials directly targeting IL-1/NF-κB in knee OA have shown limited or inconsistent benefit, supporting a shift toward upstream/mechano-immune modulation and exposure-matched designs [73]. Studies have shown that decreased interferon stimulator genes are associated with a slowdown in senescence, apoptosis, and ECM degradation triggered by IL-1β, by suppressing the NF-κB signaling axis [74]. The decrease in the M1 hairpin box protein also represses the inflammatory response induced by IL-1β in human OA chondrocytes, by inhibiting NF-κB activation [75]. Likewise, silencing of the homeobox transcription factor LIM 1 beta suppresses cellular apoptosis and inflammatory response in human OA chondrocytes induced by IL-1β via the NF-κB pathway [76]. Chondroitin sulfate, a component of cartilage, shows anti-inflammatory effects on chondrocytes and also affects the NF-κB pathway [77]. Stimulation by IL-1β leads to rapid degradation of IκBα, releasing multiple NF-κB dimers and contributing to inflammatory responses. In addition, NF-κB activation may be associated with increased intracellular oxidative stress. Under inflammatory conditions, chondrocytes undergo a metabolic shift towards aerobic glycolysis, mainly regulated by HIF-1α, which favors lactate production and alters redox homeostasis. Although the enzyme lactate dehydrogenase A (LDH-A) does not directly produce ROS, mitochondrial dysfunction and other oxidative systems contribute to the increase in ROS, which in turn can modulate and amplify NF-κB activity, perpetuating inflammation [78].

### 5.2. Nrf2 and Its Role in Antioxidant Response and Cellular Protection

Nrf2/Keap1 is the dominant antioxidant axis in osteoarthritic chondrocytes. Natural activators (e.g., curcumin, resveratrol, sulforaphane) have poor or variable bioavailability; nano-enabled formulations are, therefore, required to improve stability and exposure (and, for intra-articular use, residence time and safety) [79]. In addition to its antioxidant functions, Nrf2 plays a role in modulating inflammatory processes, in part by regulating the redox metabolism of immune cells such as macrophages. Its activation has also been associated with protective effects against OA, by reducing oxidative damage, chondrocyte apoptosis, and processes such as ferroptosis [80,81]. However, natural compounds that activate this pathway, such as curcumin, sulforaphane, and resveratrol, have important limitations, including rapid metabolization, low systemic bioavailability, and interaction with other cellular pathways [82], underscoring the need for new, more stable release systems or derivatives.

### 5.3. ADAMTS and ADAMs in the Degradation of ECM in OA

A Disintegrin and Metalloproteinase (ADAM) and A Disintegrin and Metalloproteinase with Thrombospondin Motif (ADAMTS) are key enzymes in the maintenance of organ and tissue homeostasis, but they are also involved in the pathology of OA. Inhibition of ADAM-17 is considered a promising strategy for reducing inflammation in OA [83]. In addition, several members of the ADAMTS family, such as ADAMTS-4 and ADAMTS-5, show elevated expression in joint chondrocytes, participating in the degradation of aggrecan, a component of cartilage ECM. The removal of *ADAMTS5* has demonstrated protection against cartilage destruction in animal models of OA, and specific inhibitors are being developed for potential therapeutic use. On the other hand, several ADAMs, including ADAM-8, -9, -10, -12, and -28, show overexpression in early stages of OA, while ADAM-19 and -23 are upregulated in late stages of the disease [72,84].

### 5.4. MMPs: Regulators of Cartilage Remodeling and Degradation in OA

MMPs, zinc-dependent proteolytic enzymes, play an essential role in the degradation of articular cartilage ECM in OA. They are widely distributed in connective tissues and participate in the degradation of aggrecan, a distinctive feature of OA [58]. Elevated levels of MMP13 have been observed in cartilage affected by OA and are suggested to play a central role in the irreversible degradation of type II collagen in this disease. In addition, reduction in activin-like kinase 5 (ALK5) has been associated with an increase in *MMP13* mRNA levels, indicating its involvement in the pathogenesis of OA and its potential as a therapeutic target [85]. A number of compounds, such as specific MMP-13 inhibitors and monoclonal antibodies, have been identified as potential treatments for OA [72].

### 5.5. FOXO1 in Cellular Homeostasis and Cartilage Metabolism

TGFβ signaling is critical for joint tissue development and cartilage homeostasis. Studies with transgenic mouse models have provided detailed knowledge about the key molecules in this pathway and their involvement in OA, allowing for tissue-specific, cellular, and temporal regulation [86]. FOXO1 has been found to be a mediator in articular cartilage homeostasis via the TGFβ/TAK1 pathway. Overexpression of FoxO1 in articular cartilage protects against the development of OA in models of meniscal ligament injury and OA induced by TGFβR2 loss. Autophagy is modulated by the TGFβ-FOXO1 axis through TAK1 signaling [87].

FoxO1 is mainly expressed in bone and cartilage, regulating the development of bone tissue and chondrocyte homeostasis. FoxO1 interacts with Runx2 and the Wnt/β-catenin pathway to regulate osteoblast differentiation and energy metabolism [88]. The FoxO family of transcription factors primarily includes FoxO1, FoxO3, FoxO4, and FoxO6, each with specific, overlapping functions. FoxOs are targets of PI3K/Akt signaling, which modulates cell proliferation, growth, apoptosis, and the expression of antioxidant and autophagy proteins. Autophagy and the ubiquitin-proteasome system are intracellular elimination mechanisms regulated by FoxO [89]. FoxO-mediated autophagy is essential for chondrocyte homeostasis, preventing the degradation of articular cartilage [90,91]. FoxO1 expression decreases in the cartilage of elderly patients with OA and in areas of osteophyte fibrosis and hyperplasia, correlating with OA progression [88]. FoxO1 and FoxO3 are abundant in the human menisci, and their expression decreases in the degenerated meniscus, revealing their protective functions during the progression of age-induced OA. In animal models, FoxO1 expression decreases with age and biomechanical stress-induced microstructural damage accumulates, regulating FoxO1 expression and OA progression [92].

Research on signaling pathways in OA reveals several potential therapeutic targets (Table 1). Among the molecules of therapeutic interest are IκBα, MMP-13, ADAMTS-5, Nrf2, HO-1, and FoxO1. IκBα regulates the stability of NF-κB, crucial in joint inflammation, while MMP-13 and ADAMTS-5 are key enzymes in cartilage degradation in OA. Nrf2 and HO-1 play roles in antioxidant defense and the protection of cartilage against oxidative stress. On the other hand, FoxO1 regulates chondrocyte homeostasis and the autophagic response, being essential for maintaining the integrity of cartilage in the face of oxidative stress and aging.

## 6. Therapeutic Strategies and Emerging Trends in OA and Cartilage Regeneration

### 6.1. Therapies Based on Natural Products

#### 6.1.1. Propolis: Composition, Properties, and Applications in OA

Propolis, a natural product made by bees, stands out for its biological and pharmacological properties, attributable to its complex chemical composition, which includes more than 300 compounds such as resins, waxes, aromatic oils, pollen, amino acids, minerals, sugars, phenols, and terpenes [93]. Its main biological effects derive from phenolic compounds such as phenolic acids, flavonoids, tannins, stilbenes, coumarins, and quinones. However, it faces limitations in its food use due to its low solubility in water, lack of stability, and intense taste and odor, in addition to the low bioaccessibility of its phenolic compounds after digestion [94]. The chemical composition of propolis, influenced by the flora and the place of collection, determines its biological properties, including antioxidant, anti-inflammatory, antidiabetic, antiatherogenic, antibacterial, and antifungal activities, mainly associated with its polyphenol content [95,96]. Studies with HPLC-DAD confirmed the presence of flavonoids such as quercetin, apigenin, pinocembrin, and caffeic acid phenethyl ester (CAPE) in Chilean propolis, by comparison with commercial standards and retention times [97]. Knee joints were compared in senescent rats treated with propolis (SR-EEP) and vehicle (SR-V). The results showed that in the SR-EEP group, the articular cartilage presents an improved organization, with greater thickness and articular surface, a better-quality ECM, and chondrocytes with notable proliferation and hypertrophied appearance, indicating an active repair process. In contrast, the SR-V group shows fewer structural improvements in cartilage and subchondral bone [98].

#### 6.1.2. The Role of Polyphenols in Osteoarthritis

Polyphenols play multifaceted roles in OA by dampening inflammatory signaling and catabolism while supporting cytoprotective responses. Curcumin suppresses IL-1β/OSM-induced MMP expression in human chondrocytes via NF-κB inhibition and has broad chondroprotective actions in preclinical models [99,100]. Resveratrol activates SIRT1 and inhibits NF-κB, reducing pro-inflammatory mediators and protecting cartilage [101,102]. EGCG from green tea lowers TNF-α and MMP-13 in OA chondrocytes and mitigates OA progression in vivo [103]. Quercetin attenuates OA by activating the AMPK/Nrf2/GPX4 axis and suppressing chondrocyte ferroptosis (in vitro and in vivo) [104]. Propolis-derived pinocembrin down-regulates MMP-1/-3/-13 and blocks NF-κB p65 nuclear translocation in human chondrocytes [22]. CAPE activates Nrf2/HO-1, inhibits NF-κB, and reduces iNOS/COX-2, NO, and PGE_2_ in IL-1β-stimulated chondrocytes, improving OA outcomes in vivo [105]. A concise summary of these mechanisms and outcomes is presented in Table 2. Notwithstanding these signals, many polyphenols face low solubility and poor bioavailability, motivating formulation strategies and nano-enabled delivery [106].

Clinical evidence and limitations (polyphenols). Randomized trials of curcuminoid formulations report short-term symptomatic benefit (e.g., non-inferiority to ibuprofen over 4 weeks; superiority to placebo over 6 weeks) [107,108], and a nanocurcumin micellar preparation improved WOMAC scores over 6 weeks [109]; however, samples are modest, follow-up is brief, products are heterogeneous, and structural endpoints are rarely assessed. In contrast, a phase-3 trial of oral resveratrol did not reduce knee pain versus placebo [110]. These observations support cautious optimism for select formulations while underscoring variability in bioavailability, dosing, and trial quality (See Table 3 for a concise summary of representative clinical trials).

Polyphenols, secondary metabolites of plants, are characterized by phenolic rings and hydroxyl groups that give them antioxidant properties, useful for neutralizing ROS and nitrogen (RNS) [114,115]. These reactive molecules, produced during oxidative metabolism, can induce inflammation by activating factors such as NF-κB and promoting the synthesis of pro-inflammatory cytokines such as TNF-α [116]. Polyphenols work by modulating these inflammatory processes, scavenging free radicals, inhibiting ROS-generating enzymes, and chelating metal ions such as iron and copper, which protects against oxidative damage [117]. Although their concentration is low to function as direct antioxidants, polyphenols enhance the body’s antioxidant capacity by activating the Nrf2 signaling pathway. This factor, under stress, migrates to the cell nucleus and regulates protective genes associated with AREs, which improves antioxidant defense and is linked to mitochondrial biogenesis. Studies suggest that prolonged consumption of polyphenols may increase the body’s antioxidant capacity in a similar way to the benefits obtained through physical activity [118].

Research has shown the potential of polyphenols to protect articular cartilage from degeneration. Leong et al. (2014) highlighted the effect of epigallocatechin 3-gallate from green tea in murine models, attributing its protective action to the regulation of proteoglycan homeostasis. Clinical trials also showed improvements in joint function and pain reduction following consumption of apple powder polyphenols [119]. Procyanidin B3, from grape seeds, has been reported to protect against H_2_O_2_-induced chondrocytic apoptosis and reduce the expression of iNOS in synovial tissues [120]. Other studies have noted that apple procyanidins, administered orally, could prevent OA in mice with chondrocytes-Sod2−/− under mechanical overload, improving mitochondrial biogenesis and proteoglycan synthesis [121]. However, the efficacy of these compounds depends on factors such as their concentration, chemical structure, solubility, and pharmacokinetic variability related to age, health, microbiota, diet, and previous treatments [95]. Polyphenols, natural non-enzymatic antioxidants derived from plants, have shown positive effects such as stimulating mitochondrial biogenesis and reducing oxidative stress. These compounds act by neutralizing ROS, inhibiting its production, and increasing the activity of the transcription factor Nrf2 [122]. In addition, the polyphenols present in pomegranate peel show therapeutic potential for inflammatory diseases such as OA, Alzheimer’s, inflammatory bowel disease, and cancer, by reducing the expression of inflammatory genes, the production of pro-inflammatory interleukins, and inhibiting enzymes such as NF-κB, COX-2, and iNOS [123].

#### 6.1.3. Main Bioactive Compounds: Pinocembrin and CAPE

Pinocembrin, a flavanone present in plants and products such as propolis, stands out for its ability to modulate cell metabolism and biochemical pathways, showing promising effects in inflammatory, cardiovascular and oxidative stress-related diseases. This compound activates the transcription factor Nrf2, promoting the expression of antioxidant enzymes, and has shown benefits in models of neurodegenerative diseases and cardiac dysfunction through the Nrf2/HO-1 pathway, in addition to inhibiting NF-κB signaling and advanced glycation end products [124]. In addition, its relevance in the regulation of cellular enzymatic functions and its effectiveness in the treatment of cardiovascular, anti-inflammatory, antimutagenic, and anticancer diseases are highlighted [125]. Given pinocembrin’s hydrophobicity and rapid clearance, nanoformulations developed for flavonoids, such as liposomes and polymeric micelles, can enhance solubility, stability, and intra-articular residence, providing a practical path to translate pinocembrin’s chondroprotective signals into the joint microenvironment [126].

Evidence tier: Preclinical. Pinocembrin has been shown to reduce levels of catabolic factors, such as NO/iNOS, PGE2/COX-2, and MMPs, by inhibiting the Nrf2/HO-1 pathway in human OA chondrocytes treated with IL-1β. In addition, it can suppress NF-kB signaling by inhibiting the phosphorylation of IkBα and p65, as well as by suppressing the nuclear translocation of NF-kB [24]. Two studies investigated the therapeutic potential of pinocembrin. In intervertebral disc degeneration (IDD), pinocembrin reduced cartilage endplate (CEP) calcification and disc deformation, improved cell viability, and prevented chondrocyte apoptosis; it also stimulated mitophagy via Nrf2 activation [127]. Separately, pinocembrin and caffeic acid showed anti-inflammatory properties by suppressing signaling pathways associated with MMP-9 induction in LPS-stimulated macrophages [128]. These findings support interest in pinocembrin as a promising therapeutic option to address OA. Preclinical signal is consistent for NF-κB suppression and matrix protection, but clinical plausibility is limited by uncertain intra-articular exposure and heterogeneous formulations; priority: exposure-matched dosing, joint-retention data, and pain/function plus imaging endpoints.

CAPE, a natural derivative of caffeic acid, is considered one of the most promising bioactive components present in propolis [129]. This compound was recognized as part of propolis in 1987, and its synthesis was carried out in 1988 at Columbia University. Its molecular formula is C_17_H_16_O_4_. CAPE is a polyphenol that contains hydroxyl groups in its catechol ring, which are responsible for many of its biological functions [130]. Among the biological properties of CAPE are its anti-inflammatory, antioxidant, antiviral, antibacterial, immunomodulatory, anticancer, and wound-healing activities [131].

Evidence tier: Preclinical. CAPE carries out its anti-inflammatory activities by modulating different inflammatory pathways, including inhibition of NF-κB transcription factors. CAPE inhibits NF-κB activation, suppressing IκBα degradation and p65 phosphorylation in gastric cancer cells, suggesting its therapeutic potential in inflammation. Some studies show that CAPE also inhibits NF-κB by suppressing the interaction of NF-κB with DNA, not just blocking the degradation of IκBα [132]. CAPE inhibits LPS-induced IL-12 production and NF-kB activation in monocyte-derived dendritic cells [133]. In LPS-induced breast cancer cells, CAPE can reduce the expression of TLR4, NF-kB p65, TRIF, MyD88, and IRAK4 while stimulating apoptosis and cellular autophagy [134]. In gingival fibroblasts, CAPE suppresses LPS-induced production of IL-6, IL-8, iNOS, COX-2, TLR4/MyD88-mediated NF-kB, and LPS-induced phosphorylation of PI3K and protein kinase B (PKB or Akt) [135].

To address CAPE’s poor aqueous solubility and chemical instability, β-cyclodextrin inclusion complexes and protein/peptide-based nanoparticles have been shown to increase solubility, preserve bioactivity, and improve delivery profiles, offering immediately translatable strategies that complement the SPN approach detailed below [136,137]. Preclinical signal supports antioxidant/Nrf2 activation and reduced catabolism, but stability/bioavailability and dose–exposure uncertainty limit translation; priority: formulation that improves stability and local exposure with mechanism-aligned clinical endpoints. The encapsulation of CAPE by self-assembled sorghum peptide nanoparticles (SPNs) has been studied with the aim of improving its water solubility and stability, limited by its tendency to oxidize. SPNs demonstrated high encapsulation capacity, good stability during storage, and efficient interaction with CAPE, suggesting a promising approach for application in the food and pharmaceutical industries [138].

CAPE and pinocembrin, bioactive compounds in propolis, stand out for their anti-inflammatory and antioxidant properties, which positions them as promising agents in the treatment of OA. CAPE inhibits NF-κB signaling by preventing IκBα degradation, reducing inflammation and cartilage degradation. Moreover, pinocembrin activates the Nrf2/HO-1 pathway, which counteracts oxidative stress and indirectly impacts inflammation. Its encapsulation in nanoparticles could improve its stability and bioavailability, optimizing its therapeutic use in OA.

### 6.2. Nanotechnology to Improve the Bioavailability of Polyphenols and Bioactive Compounds: Applications in OA Treatments

Although the consumption of polyphenols helps in the prevention of diseases, their oral administration without protection results in a low efficiency at the site of action. This is due to several factors, such as concentration, binding site, chemical structure, stability in the gastrointestinal environment, and aqueous solubility, which together negatively affect absorption levels, degree of metabolization, distribution in the body, shelf life, and excretion of the compound [139]. In addition, the pharmacokinetics of polyphenols are influenced by patients’ age, health status, gut microbiota, and diet, as well as oral antibiotic treatments [140]. These variables are reflected in different reports of low polyphenol bioavailability, such as 0.56–4.54 nmol/L for anthocyanins [141], 0.46–1.28 μmol/L for flavanones [142], and 37–60 nmol/L for phenolic acids [143].

Antioxidant activity and polyphenol composition in pigmented grain extracts were evaluated after digestion and transport across Caco-2 intestinal cells, showing a significant reduction in antioxidant activity after digestion [144]. Propolis-, saffron-, and curcumin-loaded ZIF-8 nanoparticles (PROZIF, SAFROZIF, CURCUZIF) showed efficient encapsulation and sustained release; in vitro analyses (SEM imaging, MTT assays, anti-inflammatory tests) indicated high therapeutic efficacy, with PROZIF exhibiting superior anti-inflammatory activity and multifaceted mechanisms, highlighting these nanocarriers as a promising alternative for OA management [145]. Protected delivery and pH-responsive release are promising strategies, but their plausibility is limited by framework stability, metal-ion safety, and regulatory familiarity. Therefore, comprehensive characterization, biodistribution studies, and immunocompatibility assessments under clinically relevant conditions should be prioritized.

Nanotechnology is presented as an optimal solution to address the limitations in the delivery and bioavailability of polyphenols, through various nano and micro encapsulation techniques. These methods include inorganic solid nanoparticles, nanometals such as gold and silver, liposomes, surfactant micelles, and extracellular vesicles. In addition, polymeric nanoparticles, various types of lipid vesicles, cyclodextrin complexes, prebiotic/probiotic-based systems, nanocrystals, and semisolid systems have been explored [146]. These nanoparticles, with variable sizes, have large surface areas that improve the solubility of bioactive compounds, making them valuable nanocarriers to improve the solubility, bioavailability, and efficacy of poorly soluble drugs [147]. Beyond improving solubility and stability, nano-systems can be engineered for chondrocyte-directed delivery and prolonged intra-articular retention; for example, chondrocyte membrane–coated nanoparticles enhanced joint residency and mitigated cartilage damage in preclinical models, providing a platform readily adaptable to phenolic payloads [148].

Nanocarriers face challenges, such as their complex chemical design, limited payload capacity, and high costs. Their widespread use as part of formulations can pose risks to patients, especially at high doses. Therefore, exploring nanocarriers with therapeutic properties could reduce the amount of drugs and carriers needed, which in turn could minimize side effects and improve treatment efficacy for patient benefit [149].

Data suggest that the solubility and local exposure of polyphenols are improved, but successful translation depends on reproducible CMC/GMP processes and long-term joint safety. Therefore, priorities should include batch reproducibility, stability assessment, biodistribution studies, and the development of a standardized chronic safety package. Among intra-articular delivery systems, triamcinolone acetonide extended-release (PLGA microspheres) has shown clinically meaningful, months-long pain reduction versus placebo and standard crystalline steroid in randomized studies [111]; diclofenac etalhyaluronate (DF-HA) has improved WOMAC pain versus placebo in phase-2/3 trials [112]; and extracellular-vesicle-based injections have shown early promise in a triple-blind randomized study [113]. Translation is advancing for certain carriers, yet long-term safety, manufacturing scalability, and regulatory pathways remain key considerations (See Table 3). While early clinical data for intra-articular delivery systems, such as triamcinolone acetonide extended-release microspheres, diclofenac-etalhyaluronate conjugates, and extracellular vesicles are promising, translation to routine care remains tempered by key challenges: limited long-term safety and potential immunogenicity with repeated dosing, the complexity of scaling manufacturing under Good Manufacturing Practice conditions with tight control of critical quality attributes, and high production costs that can affect cost-effectiveness and access. Addressing these issues proactively during preclinical and CMC development is essential to enable safe and equitable translation into osteoarthritis management [150,151,152].

### 6.3. Nanotechnology-Enhanced Delivery Systems for OA

Intra-articular (IA) platforms, including lipid, polymeric, and hybrid/inorganic systems, aim to extend joint residence and reduce systemic toxicity; clinical plausibility hinges on measured IA retention, repeat-dose joint safety, and reproducible CMC/GMP. This section focuses on IA exposure and retention, alignment with clinically used endpoints (pain, function, imaging), and manufacturability under GMP with long-term joint safety. Nanotechnology-based delivery systems have shown significant promise in augmenting the therapeutic impact of bioactive agents in OA, notably polyphenols and natural anti-inflammatory compounds. Key advantages include controlled release, targeted delivery to joint tissues, improved solubility and chemical stability, longer intra-articular residence, and decreased systemic toxicity [153,154].

#### 6.3.1. Lipid-Based Nanocarriers

Translational focus: establish joint retention and head-to-head comparisons with the same payload and demonstrate true delivery gains, together with standardized immunocompatibility and repeat-dose joint safety.

Evidence tier: Preclinical. Liposomes, due to their ability to encapsulate both hydrophobic and hydrophilic molecules, have gained traction. Recent studies demonstrated that intra-articular liposomal lubricants significantly suppress shear-stress–induced expression of catabolic genes (e.g., MMP-3, IL-1β) in preclinical OA models [155]. Moreover, a 2025 report underscores the potential of tunable liposomes in knee OA management, showing enhanced joint targeting and symptom relief. Liposomes have proven to be versatile and promising delivery systems for osteoarthritis treatment, especially for natural compounds such as glucosamine sulfate, urolithin A, triptolide, gallic acid, and resveratrol. These nanocarriers improve drug stability, bioavailability, and intra-articular residence time, providing anti-inflammatory, antioxidant, and chondroprotective effects. In preclinical models, liposomal formulations have reduced inflammation, preserved extracellular matrix components, and limited cartilage structural damage. Notably, even empty liposomes have shown benefits, highlighting their function as biolubricants. These approaches offer a promising and more natural therapeutic strategy for OA management [156]. Recent advances in liposome-based strategies for OA highlight their potential as safe and effective platforms for delivering bioactive compounds. Their ability to enhance intra-articular penetration, prolong drug release, and reduce systemic side effects positions them as a key tool in the development of more effective and sustainable therapies for this chronic disease. Liposomes improve intra-articular exposure and lubrication in preclinical OA, but clinical plausibility depends on joint-retention kinetics, repeat-dose joint safety, and CMC/GMP reproducibility.

#### 6.3.2. Polymeric Nanocarriers and Nanostructured Systems for Osteoarthritis Treatment

Preclinical evidence indicates that biodegradable polymer-based nanocarriers, such as PLGA and PEG-b-polyester micelles, have been engineered to enhance drug stability, prolong release, and improve cellular uptake. A widely cited 2019 study reported surfactant-modified polymeric nanoparticles prepared via a water/oil/water double-emulsion method, which yielded small, stable particles capable of sustained release and preserved bioactivity [157]. Although this formulation is not recent, it laid the foundation for more advanced nano-encapsulation platforms. In recent years, nanotechnology-based drug delivery systems have gained momentum due to their capacity for controlled release, increased drug stability, reduced systemic toxicity, and prolonged intra-articular retention. One innovative strategy involves the use of cyclic brush polymer (CP) nanomicelles, which consist of a hydrophobic PHEMA core and surface-grafted zwitterionic PSBMA chains. These CP nanomicelles not only demonstrated excellent lubrication in tribological tests but also encapsulated the anti-inflammatory polyphenol resveratrol (CP@RSV). In vitro analyses confirmed their high cytocompatibility, suppression of ROS, and restoration of mitochondrial function, indicating a dual mechanism based on lubrication and antioxidation [158].

Polymeric carriers enhance stability and controlled intra-articular release, but scale-up under GMP and long-term joint safety remain key gaps; priority: manufacturability at scale, degradation/by-product profiling, and standardized long-term safety.

#### 6.3.3. Hybrid and Nanocomposite Strategies

Evidence tier: Preclinical. Recent innovations target mesoporous and hybrid carriers combining multiple materials. A 2024 review on porous nanomaterials describes mesoporous silica as a versatile platform due to high loading capacity and surface functionalization options useful for OA therapies [159]. Although primarily reported in oral drug delivery contexts, Si-CD systems show potential for adaptation to intra-articular use [160].

#### 6.3.4. Metal-Based Nanoparticles

Evidence tier: Preclinical. Gold nanoparticles (GNPs) have demonstrated efficacy in modulating the gut–microbiota–joint axis, reducing osteoarthritis severity in preclinical ACLT models by shifting macrophage polarization to M2, increasing IL-10 and short-chain fatty acids like butyrate, and enhancing beneficial bacteria (e.g., Lactobacillus, Akkermansia) [161]. These mechanisms position GNPs as innovative anti-inflammatory and chondroprotective agents.

#### 6.3.5. Probiotics, Prebiotics, and Synbiotics

**Figure 3:**
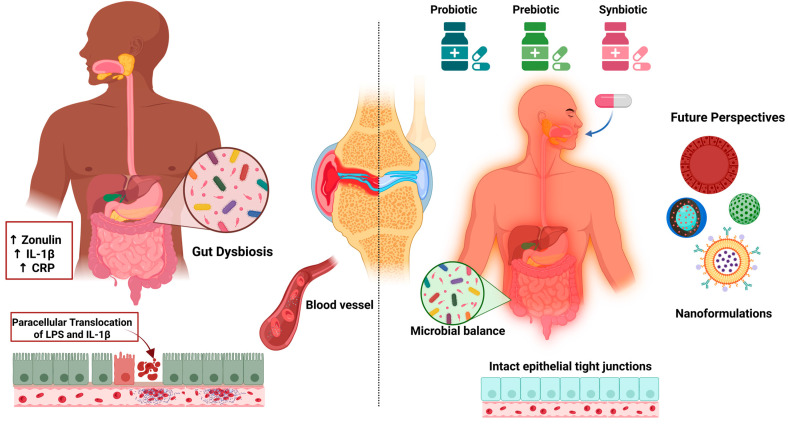
Gut dysbiosis and its role in osteoarthritis: mechanisms and therapeutic modulation using probiotics, prebiotics, synbiotics, and nanotechnology. Arrow explanation: the curved arrow indicates paracellular translocation of LPS and IL-1β across disrupted tight junctions toward the lamina propria/bloodstream; **↑** denotes increased levels. Created in BioRender. Vélez Slimani, H. (2025) https://BioRender.com/m5o9go2 (accessed on 9 September 2025).

Evidence tier: Early clinical. Growing clinical and mechanistic evidence links gut dysbiosis to the progression of OA. A 2025 meta-analysis of randomized controlled trials (n ≈ 694) demonstrated that oral probiotics significantly improved pain, joint function, and inflammatory biomarkers (e.g., hs-CRP) in patients with knee OA [162]. Previous meta-analyses have highlighted a specific benefit of *Lactobacillus* strains in reducing pain and inflammatory markers, although the overall quality of evidence remains moderate to low [163]. Although direct studies on the encapsulation of probiotics in nanoparticles are scarce, there is a strong rationale for combining probiotics or prebiotics with nanoformulations to enhance microbial viability, target delivery, and overall therapeutic outcomes [164]. Figure 3 illustrates the proposed mechanisms by which probiotics, prebiotics, and synbiotics modulate gut dysbiosis and inflammation in OA. 

A 2024 double-blind randomized clinical trial investigated the effects of a multistrain probiotic (Vivomixx^®^) in 147 elderly patients with knee OA. After 12 weeks, those in the probiotic group showed significant reductions in pain, improved postural balance and walking speed, and decreased levels of intestinal permeability markers such as zonulin, compared to the placebo group. These findings support the role of probiotics in alleviating postural imbalance in OA, potentially through modulation of the intestinal barrier and inflammatory status [165]. In another clinical trial, the strain *Latilactobacillus sakei* LB-P12 was administered to patients with chronic knee pain. After 12 weeks, participants who received the probiotic showed significant improvements in WOMAC and VAS scores, as well as enhanced quality of life. Levels of inflammatory cytokines, particularly IL-1β, were also significantly reduced. No adverse effects were reported, reinforcing the potential of targeted probiotic strains as adjunctive therapies in OA management [166].

Mechanistic studies in animal models further support that the gut microbiota plays a crucial role in bone and cartilage homeostasis. Dysbiosis has been linked not only to OA but also to other musculoskeletal conditions such as rheumatoid arthritis and osteoporosis. Dietary interventions using probiotics, prebiotics, and synbiotics have shown promise in restoring microbial balance and preventing bone and cartilage degeneration, highlighting their relevance in chronic joint disorders [167].

A recent review focusing on human orthopedic conditions concluded that modulating the gut microbiome with probiotics, prebiotics, and anti-inflammatory foods can reduce the need for NSAIDs and opioid medications [168]. This nutritional strategy offers a safer approach to managing pain and inflammation in OA and related conditions. Finally, although OA treatments remain largely symptomatic, accumulating data on the human microbiome reveals a strong link between gut dysbiosis and OA pathogenesis. While the mechanisms remain unclear, targeted microbiota modulation through probiotics, prebiotics, and synbiotics may pave the way for novel, individualized OA therapies [169].

#### 6.3.6. Translational Considerations (Regulation, CMC/GMP, Safety, Cost)

**Figure 4:**
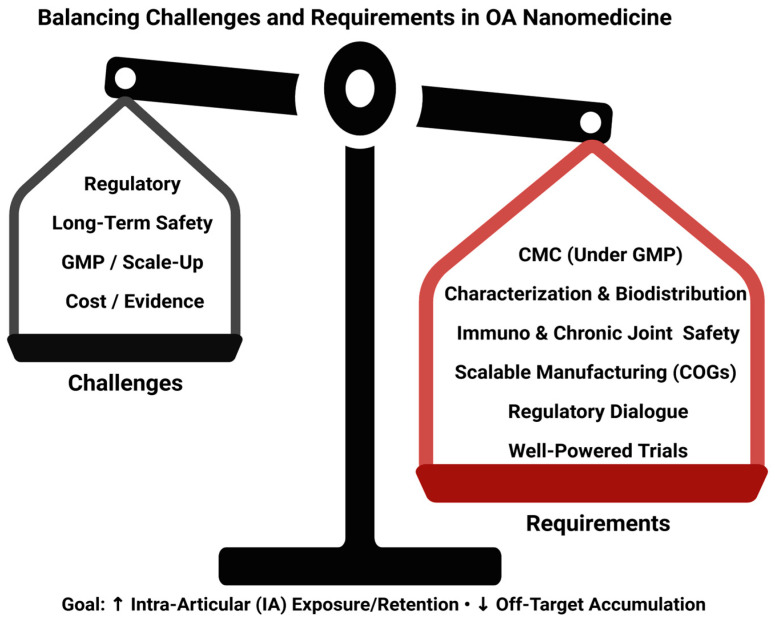
Balancing Challenges and Requirements in OA Nanomedicine. Balance schematic contrasting key challenges (regulatory, long-term safety, GMP/scale-up, cost/evidence) with requirements to enable translation (CMC under GMP, characterization & biodistribution, immune & chronic joint safety, scalable manufacturing with acceptable COGs, regulatory dialogue, well-powered trials). Goal: ↑ intra-articular (IA) exposure/retention; ↓ off-target accumulation. Abbreviations: CMC, chemistry, manufacturing, and controls; GMP, Good Manufacturing Practice; COGs, cost of goods. Created in BioRender. Vélez Slimani, H. (2025) https://BioRender.com/ptmyj90 (accessed on 9 September 2025).

Despite robust preclinical evidence and diverse nanoparticle systems under development, no nanomedicine has yet gained approval for OA therapy [170]. Regulatory expectations for nanocarriers vary across agencies and emphasize detailed physicochemical characterization and control of critical quality attributes, creating uncertainty for developers and complicating cross-region filings [150,171]. Long-term biocompatibility and potential immunogenicity, especially with repeated intra-articular administration, require dedicated chronic safety studies and standardized assays [172]. Reproducible, scalable manufacturing under GMP remains a bottleneck due to batch-to-batch variability and process sensitivity at the nanoscale [152]. Finally, high production costs and limited head-to-head data raise questions about cost-effectiveness versus existing options. Progress will depend on harmonized regulatory frameworks, robust CMC platforms, and well-powered clinical trials that include structural endpoints and longer follow-up (Figure 4).

Beyond proof-of-concept efficacy, nano-enabled platforms must demonstrate batch reproducibility, stability, and impurity control (CMC/GMP); provide comprehensive characterization and biodistribution; and demonstrate immunocompatibility and long-term joint safety. Scalability and cost-of-goods are critical for chronic OA care. Early regulatory dialogue and harmonized in vivo safety packages (e.g., complement activation and hemocompatibility) increase the probability of advancing to late-stage trials. Platforms that improve local exposure/retention with minimal off-target accumulation and can be manufactured reproducibly at scale are the most likely to progress.

## 7. Perspective and Research Priorities

This review integrates the molecular rationale for polyphenols with the engineering levers of nanocarriers under a single translational lens. Beyond summarizing signals, we align mechanisms, exposure, and endpoints to clarify what is needed for clinical progress.

Research priorities. We prioritize quantifying synovial-fluid and tissue pharmacokinetics in large-animal osteoarthritis models with phenolic payloads and adding imaging readouts for joint residency; aligning mechanisms to clinically meaningful endpoints by pairing pain and function with MRI measures of cartilage and bone-marrow lesions and by including soluble biomarkers that track NF-κB/Nrf2 activity; and standardizing dose and formulation reporting, including CMC attributes, release kinetics, and batch variability, to enable reproducible dosing across studies. Additional priorities are to assemble a harmonized safety package covering immunocompatibility, complement activation, hemocompatibility, repeat-dose joint safety, and off-target biodistribution; to conduct head-to-head platform comparisons using the same payload and dose to identify true delivery gains; and to assess scalability and cost by modeling cost-of-goods and manufacturability for chronic intra-articular use and by defining target product profiles.

## 8. Approach to Literature Selection

A narrative literature review was conducted through a comprehensive search of PubMed, Scopus, Web of Science, SciELO, and Google Scholar, covering publications up to July 2025. Keyword combinations such as “osteoarthritis,” “articular cartilage,” “chondrocytes,” “oxidative stress,” “inflammation,” “polyphenols,” “pinocembrin,” “CAPE,” “β-cyclodextrin,” “porous silicon,” “nanoparticles,” and “controlled release systems” were used, applying Boolean operators AND and OR to refine the results. The review included in vitro and in vivo studies addressing osteoarthritis pathophysiology, as well as those focused on therapeutic strategies based on natural compounds, nanomaterials, or drug delivery technologies. Studies on the synthesis and characterization of β-cyclodextrin-functionalized porous silicon nanoparticles were also considered. Exclusion criteria included duplicate records, studies without full-text access, publications in languages other than English or Spanish, and articles lacking relevant experimental evidence. In addition, structured databases such as PubChem were consulted for physicochemical data of the compounds studied, and digital tools such as BioRender were used to generate scientific illustrations. All included studies were critically evaluated based on methodological quality, recency, and scientific relevance. Evidence tiers. We classify evidence as Preclinical (in vitro or animal studies), Early clinical (pilot or early-phase trials), and Late clinical (adequately powered randomized or confirmatory trials). These labels are used throughout the manuscript to indicate the maturity of evidence. We labeled each subsection with the predominant evidence tier (Preclinical, Early clinical, Late-stage clinical) and added an ‘Evidence level’ column to Table 3 to make the maturity of the evidence explicit.

## 9. Critical Appraisal: Why Preclinical Signals Fade in Clinical Trials

Although polyphenols and nano-enabled delivery consistently modulate inflammatory and oxidative pathways in chondrocytes and small-animal OA models, these signals rarely translate into robust clinical benefits. Three factors mainly drive this gap: exposure mismatches, effective in vitro concentrations often exceed intra-articular or systemic levels achievable with safe dosing; model limitations, surgical or cytokine-driven models privilege short-term surrogates and do not capture structural progression or patient-relevant function; and endpoint mismatch, biochemical readouts (e.g., MMPs, GAG release) map poorly onto pain, function, and imaging progression used in trials. Closing this gap requires exposure-matched designs, standardized models with structural/functional outcomes, and mechanism-aligned clinical endpoints. Whenever possible, preclinical studies should report dose–exposure relationships, joint retention, and batch/formulation variability to better inform trial design.

## 10. Conclusions

OA remains a multifactorial and progressive joint disorder with no approved disease-modifying therapies to date. This review highlights the therapeutic promise of natural compounds, particularly polyphenols derived from propolis, such as pinocembrin and caffeic acid phenethyl ester, which exhibit significant anti-inflammatory, antioxidant, and chondroprotective properties. These bioactive agents modulate key molecular pathways implicated in OA progression, including NF-κB, and oxidative stress mediators. Nanotechnology has emerged as a powerful platform to overcome limitations associated with poor solubility, chemical instability, and short intra-articular retention of natural compounds. Advanced delivery systems such as liposomes, polymeric micelles, hybrid nanoparticles, and cyclodextrin-functionalized nanocarriers have demonstrated enhanced tissue targeting, sustained release, and improved therapeutic outcomes in preclinical models. In addition, metallic nanocarriers and biomimetic coatings are being explored as innovative approaches to optimize pharmacological delivery in OA. Concurrently, growing evidence supports the gut–joint axis as a therapeutic target. Probiotics, prebiotics, and synbiotics have shown potential to restore gut microbiota balance, reduce systemic inflammation, and improve clinical symptoms in OA patients. These strategies offer a complementary avenue that may enhance therapeutic efficacy through immunometabolic modulation. Taken together, current advances underscore a paradigm shift from conventional symptomatic management toward multi-targeted therapeutic strategies that integrate natural compounds, microbiota modulation, and nanotechnology-based drug delivery. Further clinical studies are essential to validate their long-term safety, efficacy, and translational applicability in the context of OA.

## Figures and Tables

**Table 1:** Molecules involved in OA.

Signaling Pathway	Description	References
IkBα	It regulates the stability of NF-κB, related to joint inflammation.	[51,52,53,54,55,62]
MMP-13	Key enzyme in the degradation of type II collagen.	[62,63,64]
ADAMTS-5	An enzyme involved in the degradation of aggrecan in cartilage.	[61,62]
Nrf2	Regulates cellular antioxidant response.	[57,58]
HO-1	Protects cartilage against oxidative stress.	[57,58]
FoxO1	It regulates chondrocyte homeostasis and autophagic response.	[69,70]

**Table 2:** Representative polyphenols, key pathways, and OA-relevant outcomes.

Polyphenols	Key Pathways/Targets	OA-Relevant Outcomes	Ref.
Curcumin	NF-κB ↓; MMPs ↓	Suppresses IL-1β/OSM-induced MMPs in chondrocytes; chondroprotection	[95,96]
Resveratrol	SIRT1 ↑; NF-κB ↓	Anti-inflammatory; protects cartilage	[97,98]
EGCG	NF-κB/TNF-α ↓; MMP-13 ↓	Lowers TNF-α and MMP-13; slows OA progression (in vivo)	[99]
Quercetin	AMPK/Nrf2/GPX4 ↑	Inhibits chondrocyte ferroptosis; in vitro/in vivo benefit	[100]
Pinocembrin	NF-κB (p65) nuclear translocation ↓; MMP-1/-3/-13 ↓	Anti-catabolic; chondroprotection	[101]
CAPE	Nrf2/HO-1 ↑; NF-κB ↓; iNOS/COX-2 ↓	Reduces NO and PGE_2_; in vitro + in vivo improvement	[102]

Symbols: ↑ increase/upregulation; ↓ decrease/downregulation (relative to control/untreated).

**Table 3:** Representative clinical trials of polyphenols and nanomedicine-based interventions in knee osteoarthritis.

Category	Study (Year)	Design/N	Intervention (Dose; Duration)	Comparator	Primary Outcome	Main Limitations	Evidence Tier
Polyphenol	*Curcuma domestica* extract (2014) [107]	RCT, non-inferiority; *n* = 367	1500 mg/day; 4 weeks	Ibuprofen 1200 mg/day	Non-inferior pain/function; fewer GI adverse events	Short duration; single country; product heterogeneity	Early clinical
Polyphenol	Curcuminoids (2014) [108]	RCT, double-blind; *n* ≈ 40	1500 mg/day; 6 weeks	Placebo	↓ WOMAC pain/stiffness/function	Small sample; short follow-up; product variability	Early clinical
Polyphenol (nano)	Nanocurcumin (2020) [109]	RCT, double-blind; *n* ≈ 70	40 mg q12h; 6 weeks	Placebo	↓ WOMAC total and subscales	Short duration; single center	Early clinical
Polyphenol	Resveratrol (2024) [110]	Phase 3 RCT; *n* ≈ 140	40 mg bid → 20 mg bid; 6 months	Placebo	No reduction in knee pain (primary endpoint)	Underpowered; concomitant medications	Late clinical (negative)
Nanomedicine	TA-ER (PLGA microspheres) (2018) [111]	Phase 3 RCT/analyses	IA 32 mg single dose; follow-up to 24 weeks	Saline placebo/TA crystalline	Greater, durable pain relief vs. comparators	Symptomatic steroid; generalizability; post hoc data	Late clinical
Nanomedicine	Diclofenac etalhyaluronate (2021) [112]	Phase 2/3 RCTs	IA 30 mg every 4 weeks; 12–24 weeks	Placebo	↓ WOMAC pain vs. placebo	Regional development; long-term safety pending	Late clinical
Nanomedicine	MSC-derived extracellular vesicles (2024) [113]	RCT, triple-blind	IA EVs (protocol-defined); short-term follow-up	Saline placebo	Improved pain/function; acceptable short-term safety	Early phase: manufacturing/standardization challenges	Early clinical

Evidence tiers: Preclinical = in vitro or animal studies; Early clinical = pilot or early-phase trials; Late clinical = adequately powered randomized or confirmatory trials. “Late clinical (negative)” indicates a late-stage study with a non-significant primary endpoint. Symbols: ↓ decrease/downregulation (relative to control/untreated).

## Data Availability

No new data were created or analyzed in this study. Data sharing is not applicable to this article.

## Abbreviations

The following abbreviations are used in this manuscript:

ADAMTS	A Disintegrin and Metalloproteinase with Thrombospondin Motifs
ARE	Antioxidant Response Element
CAPE	Caffeic Acid Phenethyl Ester
CCL	Calcified Cartilage
CD	Cyclodextrin
COX-2	Cyclooxygenase-2
ECM	Extracellular Matrix
GAG	Glycosaminoglycans
GST	Glutathione S-transferase
HO-1	Heme Oxygenase-1
IL-1β	Interleukin-1 beta
IL-6	Interleukin-6
MMP	Matrix Metalloproteinase
NF-κB	Nuclear Factor kappa B
NO	Nitric Oxide
NSAIDs	Nonsteroidal Anti-inflammatory Drugs
OA	Osteoarthritis
PGE2	Prostaglandin E2
RER	Rough Endoplasmic Reticulum
ROS	Reactive Oxygen Species
TNF-α	Tumor Necrosis Factor-alpha
CMC	Chemistry, Manufacturing, and Controls
GMP	Good Manufacturing Practice
COGs	Cost of Goods
IA	Intra-Articular
BD	Biodistribution
